# Ontario Veterinary College

**Published:** 1890-05

**Authors:** 


### VETERINARY COLLEGE NOTES.

*Ontario Veterinary College.*—The Board of Examiners for the Province of Ontario met in the upper lecture room of the Ontario Veterinary College, on the 31st of March. The following veterinary surgeons, members of the board, were present, viz.: Messrs. Wilson, of London; Sweetapple, of Toronto; Cowan, of Galt; Elliott, of St. Catharine's; Loyd, of New-market; O'Neil, of London; Shaw, of Dayton, Ohio, U. S.; Wm. S. Thorburn, of Toronto. A large number of students went up to be examined for graduation, of whom 140 succeeded in passing.

On the 5th of April there was a gathering of students and friends of the college to hear the results of the examinations read, the honors and prizes being awarded at the same time. The assemblage took place in one of the commodious lecture halls of the college.

Upon the platform were seated the chairman, Professor Smith, F.R.C.V.S.; Sir Daniel Wilson, Hon. C. A. Pope, U. S. Consul; Dr. Thorburn, Dr. May, Dr. Aikins, Dr. Duncan, Dr. O'Reilly. Principal Cavan, of Knox College; Professors Ramsay, Wright, G. C. Richardson and Loudon, Alderman Frankland, Alderman Dodds and others. The chairman opened the proceedings with a brief account of the college over which he presides, and which owes so much of its efficiency to his zeal and ability. At the conclusion of his address the list of graduates, with the prize and honor list, was read. Presentation of the prizes followed, and many of the distinguished guests took part in this ceremony, making short congratulatory remarks upon the work which the Ontario Veterinary College has accomplished, as evidenced by its roll of 700 graduates, and by the large and increasing attendance upon the teaching of the principal and his assistants. Sir Daniel Wilson, President of Toronto University, in the course of his remarks, bore brief, but emphatic testimony to the high standing and efficiency of the college, and heartily congratulated Principal Smith on the success of his untiring efforts on behalf of veterinary medical education. Dr. Aikins, dean of the Faculty of Medicine in Toronto University, referred in eulogistic terms to the excellent arrangements for lighting and ventilating the lecture rooms of the college. Hon. C. R. Pope, in presenting the gold medal awarded to the student passing the best examination of the year (J. H. Ullrich, of Decatur, Illinois), made a speech instinct with eloquence and irradiated with flashes of genuine humor. Referring to the large attendance of American students, he considered that this was an evidence of their acumen, as they were sharp enough to know a good thing when they saw it. Alderman Frankland made a characteristic and happy speech, and Alderman Dodds referred to the wide distribution of the graduates of this college. Within the past few days he had received letters from a town on the Atlantic coast, from Honolulu, Sandwich Islands, and from his own son, who was practicing in the city of Cincinnati, and the writers were all doing excellent practices, showing the good practical training given in the Ontario Veterinary College.

After the presentation of prizes had been concluded, Mr. W. I. Broaddus, of the graduating class, came forward, and, in a clever speech, begged the acceptance, by Professor Smith, of a large class photograph of the graduates as a slight token of their esteem for him.

Professor Smith, in accepting the handsome gift, feelingly referred to the happy relations always existing between himself and the students, and most heartily wished them all possible success in their life-work.

Following is the list of graduates and the honor list :—

J. T. Arrell, Caledonia, Ont.; George E. W. Ashton, North Shefford, P. Q.; William J. Armour, Milton, Ont.; W. G. E. Austin, Ottawa, Ont.; S. W. Bailey, Millbury, Ohio, U. S.; Samuel A. Bell, Watertown, N. Y., U. S.; Edmond E. Bittles, Waterford, Pa., U. S.; Willis Grant Benner, Allentown, Pa., U. S.; Orville L. Boor, Newcastle, Ind., U. S.; Bruce G. Brake, St. Thomas, Pa., U. S.; S. Broad, Little Britain, Ont.; A. T. Brodie, Almira, Ont.; William H. Bucke, West Manchester, Ohio, U. S.; Edgar Burwell, South Edgar, Pa., U. S.; Will L. Broaddus, Connersville, Ind., U. S.; Frank Bales, Dana, Ind., U. S.; Joseph Carr, Elm Grove, Ont.; H. G. Carnes, Greenwood, Ind., U. S.; William R. Carr, Westfield, Ont.; Charles H. Clark, Grand Rapids, Michigan, U. S.; Peter Cook, Clinton, Ont.; Robert H. Cook, Malton, Ont.; Robert E. Cooper, Corvallis, Oregon, U. S.; Walter D. Cowan, Galt, Ont.; Michael Henry Davitt, Springfield, Mass., U. S.; James Donoho, Ridgetown, Ont.; William Dudgeon, Orangeville, Ontario; James W. Darby, Dalesboro, Assa.; William B. Elliott, Greenock, Ont.; John W. Elliott, Aberdeen, S. Dak., U. S.; James C. Elviage, Delaware, Ont.; Richard W. Evans, Coldstream, Ont.; Aubrey H. Fitch, Niagara Falls South, Ont.; James Fleming, Detroit, Mich, U. S.; U. Grant Fridirici, Tamaqua, Pa., U. S.; Philip Francis Finnigan, Syracuse, N. Y., U. S.; J. Johnson Fyle, Brantford, Ont.; W. J. Gerrow, Omemee, Ont.; John R. Giffen, Mayfield, Ont.; W. G. Gilpin, Ottawa, Ont.; Joseph Golley, Wingham, Ont.; Lonzo B. Good, Columbus Grove, Ohio, U. S.; J. Ross Hale, Upper Sandusky, Ohio, U. S.; Philip Herold, Fairberry, Neb., U. S.; Arther C. Hart, Richfield, Ohio, U. S.; P. Heseltine, Colborne,Ont.; F. A. Heslop, Appleby, Ont.; Jesse Z. Hillegass, Pennsburg, Pa., U. S.; Charles C. Hooker, Dana, Ind., U. S.; C. B. Horr, Greenwich, Ohio, U. S.; A. L. Hoisington, Canaan, Ohio, U. S.; Charles E. Hatch, Gainesville, N. Y., U. S ; S. A. Ireland, Laskay, Ont.; Samuel Irwin, Orangeville, Ont.; George Jobson, Franklin, Pa., U. S.; Thomas Johnston, Bailieboro', Ont.; W. G. Jones, Strathroy, Ont.; Cornelius B. Jones, Chillicothe, Ohio, U. S.; Charles Jose, Halloway, Ont.; Spencer J. Jupp, Petrolia, Ont.; Charles A. Keene, Providence, R. I., U. S.; William W. Kennedy, Woodstock, Ont.; George Kerr, Mitchell, Ont.; Frank J. King, Uxbridge, Ont.; William Lawson, Walkerton, Ont.; W. B. Lein, Elmira, Ont.; John L. Linkwiler, Hillsboro, Ill., U. S.; Wm. Little, Glenora, Man.; W. S. Lyons, Markdale, Ont.; J. Gordon McPherson, Elgin, Oregon, U. S.; Dennis McCallister, Kendall, Ont.; Robert W. McCully, St. Thomas, Ont.; James McDonald, Summerside, P. E. I.; William H. McEvers, Chicago, Ill., U. S.; Christopher McRae, Alexandria,

Ont.; J. M. McKay, Malton, Ont.; J. N. McLean, Alliston, Ont.; Otis H. Mohney, Vicksburg, Mich., U. S.; Peter Malcolm, St. Mary's, Ont.; Wallace E. Martin, Toronto, Ont.; Wesley A. Mann, Strathroy, Ont.; Benjamin H. Merchant, Arcadia, Wis., U. S.; Charles Rodger Mitchell, Shirgley, Ont.; Charles S. Moore, Wellsville, N. Y., U. S.; Frank Morrow, Strathroy, Ont.; John Alfred Mowbray, Whitby, Ont.; Andrew L. Milroy, Covington Centre, N. Y., U. S.; F. W. Menhennitt, Champion, Mich., U. S.; J. W. Nagle, Salford, Ont.; E. S. Noble, Blair, Neb., U. S.; J. L. Oille, St. Thomas, Ont.; Malcolm Oliver, Stayner, Ont.; H. N. Osler, Lincoln Falls, Pa, U. S.; J. O'Connor, Freelton, Ont.; Hugh S. Orr, Canaan, Ohio, U. S.; D. F. Parker, London, Ont.; R. M. Pardue, Sacramento, Cal., U. S.; Fred W. Philp, Sparta, Ont.; B. C. Pilkey, Lindsay, Ont.; John James Pink, Hull, P. Q.; William Routledge, London, Ont.; Edwin C. Radley, Chatham, Ont.; R. M. Raban, Bristol, England; Albert E. Ramsay, Edon Mills, Ont.; William J. Regan, Orillia, Ont.; Edward Rittich, Germantown, Ohio, U. S.; James Ritchie, Brechin, Ont.; A. Robertson, Elliott, Ont.; Daniel H. Rowe, Consecon, Ont.; Daniel Ryder, Chambersburg, Pa., U. S.; John A. Routhier, Ottawa, Ont.; Clifford B. Rowell, Fairport, N. Y., U. S.; Warner B. Scott, Paddy's Run, Ohio, U. S.; William V. Sharp, Brookton, N. Y., U. S.; Philip K. Sidebottom, Manchester, England; H. W. Skerritt, Deansville, N. Y., U. S.; Alfred W. Swedberg, Sterling, Va., U. S.; Erland D. F. Smith, West Richfield, Ohio, U. S.; James M. Smith, Cherokee, Iowa, U. S.; James H. Spence, St. Mary's, Ont.; Mahlon G. Stover, Coopersburg, Pa., U. S.; Charles M. Strickler, Greencastle, Pa., U. S.; George R. Switzer, Woodham, Ont.; John C. Stewart, Danville, Ill., U. S.; John H. C. Todd, Goodwood, Ont.; George Robert Teeple, Napoleon, Ohio, U. S.; Addison J. Terry, Ferrytown, Pa., U. S.; James R. Thorne, Simcoe, Ont.; William H. Turner, Lodie, Ohio, U. S.; Thomas Telfer, Lowville, Ont.; John H. Ullrich, Decatur, Ill., U. S.; Thomas M. Waldron, Greensburg, Pa, U. S.; J. N. Walton, Woodstock, Ont.; O. G Whitestine, Huntington, Ind, U. S.; John H. Wilson, London, Ont.; Egerton T. Wilson, Shetland, Ont.; William C. Wootton, Wellman's Corners, Ont.; Leslie A. Wright, Winfred, Dak., U. S.; Alexander Wannan, Kendall, Ont.; E. T. Lawley-York, Buffalo, N. Y., U. S.

PRIMARY EXAMINATIONS.

Those who passed the primary examinations were: *Materia Medica*—Newton Cossitt, G. M. Hodgins, James A. Kelley, Lyman D. Lockwood, Adam McMillan, S Mathers, Charles Pink.

*Anatomy*.—Thomas F. Arnold, J. H. Cornell, William Leslie, David Lewis, John F. Milne, C. L. Peck, G. R. Scott, John B. Sowers, Albert E. Taylor, Norman Thomson.

THE PRIZE LIST.

Following is the complete list of prize-winners:

*Pathology*—Seniors—Leslie Wright, silver medal; Will I. Broaddus, J. H Ullrich, equal, second; P. R. Sidebottom, third. Honors—T. Arrell, S. H. Bailey, Frank Bales, W. G. Benner, O. L. Boor, H. Carnes, C. H. Clark, L. M. Cook, R. E. Cooper, W. R. Carr, H. M. Davitt, J. W. Elliott, A. H.

Fitch, F. J. Fyle, A. L. Hoisington, J. R. Hale, C. B. Horr, S. A. Ireland, W. G. Jones, Spencer Jupp, G. Jobson, I. L. Linkwiler, J. M. McKay, R McCully, B. H. Merchant, Frank Morrow, J. A. Mowbray, E. S. Noble, J. L. Oille, A. E. Ramsey, W. Regan, D. R. Rowe, W. V. Sharpe, J. M. Smith, G. R. Teeple, A. E. Terry, J. N. Walton, E. T. Wilson, W. C. Wootton, E. T. L. York.

*Materia Medica.*—Seniors—John H. Ullrich, first prize ; W. I. Broaddus, second ; L. A. Wright, third. Honors—H. G. Carnes, W. G. Jones, J. L. Oille, E. T. L. York, M. H. Davitt, J. M. McKay, P. R. Sidebottom.

*Chemistry.*—Seniors—John H. Ullrich, first prize ; J. McKay, second ; J. J. Fyle, third prize. Honors—J. T. Arrell, R. H. Cook, J. McDonald. W. J. Rogers, A. W. Swedborg, R. E. Cooper, M. H. Davitt, F. J. King, H. W. Skerritt, L. A. Wright.

*Morbid Anatomy*—Seniors.—John H. Ullrich, first prize, a set of Darwin's works, presented by Mr. W. Christie, trustee of Toronto University ; R. E. Cooper, second ; L. A. Wright and R. Cook, equal, third prize. Honors—W. G. Benner, A. C. Hart, C. B. Rowell, W. C. Wootton, M. H. Davitt, P. Malcolm, D. H. Rowe.

*Anatomy.*—Seniors—W. I. Broaddus, silver medal ; J. H. Ulrich, second prize ; W. C. Wootton, third prize. Honors—F. B. Bales, W. G. Benner, H. Carnes, R. Cook, M. H. Davitt, W. G. Gilpin, J. R. Hale, S. A. Ireland, S. Jupp, W. W. Kennedy, J. M. McKay, F. Morrow, E. T. Noble, F. W. Philip, W. V. Sharpe, H. W. Skerritt, A. J. Terry, E. T. L. York, S. Bell, O. L. Boor, C. H. Clark, R. E. Cooper, J. J. Fyle, J. Golley, A. C. Hart, G. Jobson, J. A. Kelley, W. Lawson, B. H. Merchant, C. R. Mitchell, J. L. Oille, D. H. Rowe, P. R. Sidebottom, G. R. Teeple, L. A. Wright.

*Dissected Specimens.*—Seniors—Gold medal given by the Toronto Industrial Exhibition Association awarded to Leslie A. Wright, of Winfred, Dakota, U. S.; J. W. Elliott, second prize, $30 ; R. M. Raban, third prize, $20 ; H. W. Skerritt, fourth prize.

*Entozoa.*—Seniors—Leslie A. Wright, first prize. Honors—J. T. Arrell, S. Broad, M. H. Davitt, J. J. Fyle, J. Golley, C. B. Horr, G. Jobson, R. McCully, B. H. Merchant, J. O'Connor, G. R. Teeple, O. L. Boor, R. Cook, A. H. Fitch, W. G. Gilpin, A. C. Hart, S. A. Ireland, W. W. Kennedy, J. McDonald, J. A. Mowbray, A. E. Ramsay, W. C. Wootton.

*Physiology.*—Seniors—Robert H. Cook, silver medal ; John H. Ullrich, second prize ; W. C. Wootton, third prize. Honors—J. T. Arrell, W. J. Broaddus, Frank Bayles, J. J. Fyle.

*Best General Examination.*—Gold medal given by the Ontario Veterinary Medical Association, awarded to John H. Ullrich, of Decatur, Illinois, U. S. Honors—W. G. Benner, W. J. Broaddus, J. J. Fyle, J. A. Mowbray, G. R. Teeple, L. A. Wright, O. L. Boor, Henry G. Carnes, C. B. Jones, Philip K. Sidebottom, A. J. Terry.

*Pathology.*—Juniors—George Robb, first prize ; H. Sisson, second prize; H. F. Vulliany, third prize ; Jackson Meadows, H. M. Batchelder, J. O. F. Price, James A. Pendergast, V. W. Shirley, fourth prize. Honors—David E. Augustin, H. M. Balchelder, James T. Boothby, Hamilton Bewell, Samuel G. Burkholder, W. M. Burdeck, W. E. Carnes, E. J. Cobleigh, Wilson Corliss,

Eli M. Crawford, W. F. Crewe, A. Crowforth, L. E. Day, Joseph Dietz, James Drury, Thomas E. Early, Charles E. Edmonds, James E. Everist, George Fitzgerald, J. E. Foster, C. Galbraith, John A. Genung, J. M. Gibb, Charles Hackett, John M. Hagermann, D. C. Hanawalt, John A. Jobson, J. E. King, A. D. Lamberson, W. B. McCurdy, Alex. Machan, H. L. Marsack, Jackson Meadows, George W. Moore, Thomas Morrissey, W. A. Nixon, James A. Pendergast, LaRue Philp, E. C. Porter, Joseph E. Hodgins, E. E. Holmes, A. G. Hopkins, J. O. F. Price, Jackson Rhodes, C. Sham, V. W. Shirley, R. H. Smith, S. L. Teeple, Joseph A. Thompson, Samuel Thompson, S. F. Tolmie, W. G. Turner, J. A. Hanisch, E. A Hurkley, Ernst J. Walsh, Jacob Wilhelm.

*Anatomy.*—Juniors—S. G. Burkholder, silver medal; J. G. Boothby, second prize; W. J. Wadsworth, J. A. Jobson, S. Sisson, equal, third prize. Honors—A. T. Augustine, P. Bowlby, W. F. Crewe, W. Corlis, L. E. Day, J. C. Everist, J. E. Foster, A. G. Hopkins, J. E. Hodgins, M. B. McCurdy, J. A. Pendergast, J. R. Roades, W. S. Stinson, C. Shaird, S. Thomson, A. J. Walsh, H. M. Batchelder, H. Bowen, W. E. Carnes, E. J. Cobleigh, James Drury, A. Findlay, Clark Galbraith, E. B. Holmes, A. J. Lamberson, G. W. Moore, E. C. Porter, George Robb, V. W. Shirley, S. T. Teeple, H. S. Vulliany.

*Physiology.*—Juniors—E. H. Holmes and S. Sisson, equal, first prize; J. A. Jobson, second prize; H. F. Vulliany, third prize. Honors—James Downy, G. W. Moore, J. M. Gibb, R. H. Smith.

*Chemistry.*—Juniors—A. G. Hopkins, first prize; J. M. Gibb, second prize; H. Marsack, third prize. Honors.—J. Drury, E. B. Holmes, G. W. Moore, R. H. Smith, H. F. Vulliany, J. E. Foster, R. A. McLoughery, Jas. O. Pendergast, A. J. Sanderson.

